# Dabrafenib Promotes Schwann Cell Differentiation by Inhibition of the MEK-ERK Pathway

**DOI:** 10.3390/molecules26082141

**Published:** 2021-04-08

**Authors:** Kyuhee Park, Yoonkyoung Shin, Gyeongbeen Lee, Hwantae Park, Yongmun Choi

**Affiliations:** 1 Biocenter, Gyeonggido Business and Science Accelerator, Suwon 16229, Korea; qheepark@gbsa.or.kr (K.P.); luckybin@gbsa.or.kr (G.L.); 2 Department of Physiology, Peripheral Neuropathy Research Center, Donga University Medical School, Busan 49201, Korea; sykpooh@dau.ac.kr (Y.S.); phwantae@dau.ac.kr (H.P.)

**Keywords:** Schwann cell, dabrafenib, differentiation, ErbB2, ERK

## Abstract

Schwann cell differentiation involves a dynamic interaction of signaling cascades. However, much remains to be elucidated regarding the function of signaling molecules that differ depending on the context in which the molecules are engaged. Here, we identified a small molecule, dabrafenib, which promotes Schwann cell differentiation in vitro and exploited this compound as a pharmacological tool to understand the molecular mechanisms regulating Schwann cell differentiation. The results indicated that dabrafenib inhibited ERK phosphorylation and enhanced ErbB2 autophosphorylation and Akt phosphorylation, and the effects of dabrafenib on ErbB2 and Akt phosphorylation were phenocopied by pharmacological inhibition of the MEK-ERK signaling pathway. However, the small molecule inhibitors of MEK and ERK had no effect on the expression of Oct6 and EGR2, which are key transcription factors that drive Schwann cell differentiation. In addition, pharmacological inhibition of phosphatidylinositol-3-kinase (PI3K) almost completely interfered with dabrafenib-induced Schwann cell differentiation. These results suggest that the ErbB2-PI3K-Akt axis is required for the induction of Schwann cell differentiation by dabrafenib in vitro. Although additional molecules targeted by dabrafenib remain to be identified, our data provides insights into the crosstalk that exists between the MEK-ERK signaling pathway and the PI3K-Akt axis in Schwann cell differentiation.

## 1. Introduction

In the adult peripheral nervous system (PNS), mature Schwann cells wrap nerve fibers similar to the oligodendrocytes in the central nervous system [1]. However, unlike oligodendrocytes, Schwann cells can support axon regeneration upon nerve injury in the PNS. This is primarily attributable to Schwann cell plasticity, which is described as the intrinsic capacity of mature Schwann cells to dedifferentiate into proliferative progenitor-like cells upon axon degeneration [2]. The dedifferentiated cells produce neurotrophic factors that promote the survival of injured axons and axonal elongation. Following axonal regeneration, the progenitor-like cells differentiate again into myelinating Schwann cells to support the functional recovery of peripheral nerves [3].

Recent years have seen some progress in understanding the molecular mechanisms underlying Schwann cell plasticity, and a range of signaling molecules involved in the dedifferentiation and redifferentiation of Schwann cells have been identified [4]. Among these is the c-Jun transcription factor, which is highly upregulated in Schwann cells following nerve injury and functions to induce a demyelination program [5]. The expression of c-Jun is downregulated as Schwann cells undergo redifferentiation, which is induced by axonal signals including the interaction of neuregulin 1 (Nrg1) type III in the regenerated axons and its receptors (ErbB2/3) [6,7]. As a result, the ErbB2/3 receptor tyrosine kinase and possibly other signaling pathways trigger the expression of genes required for the differentiation of Schwann cells and myelination. The transcription factors Oct6 and EGR2 are central figures in these signaling pathways. Although genetic and biochemical studies have uncovered intrinsic mechanisms linking axonal signals to Schwann cell differentiation, the activity and function of signaling molecules that differ depending on developmental stages of Schwann cells need to be determined [8].

Bioactive small molecules are valuable tools for exploring complex cellular processes [9,10]. We envisioned that identifying small molecules and elucidating molecular mechanisms for small molecules would exploit and induce Schwann cell differentiation, thus providing insight not only into the molecular events in cells undergoing differentiation but also into the development of biomolecules controlling the Schwann cell plasticity. In this study, our efforts to discover such small molecules led to the identification of dabrafenib, and by exploiting dabrafenib as a pharmacological tool, we provide further evidence regarding the role of the MEK-ERK pathway in negatively regulating Schwann cell differentiation in vitro.

## 2. Results

### 2.1. Identification of Dabrafenib That Promotes Schwann Cell Differentiation In Vitro

In an effort to discover small molecules that induce Schwann cell differentiation in vitro, we used a biomarker for Schwann cell differentiation, Oct6, in cell-based screens; the Oct6 expression level increases during Schwann cell differentiation. Indirect immunofluorescence and Oct6 quantitation in rat primary Schwann cells treated with either DMSO or compounds enabled our ability to evaluate a compound to induce Schwann cell differentiation in vitro.

**Figure 1:**
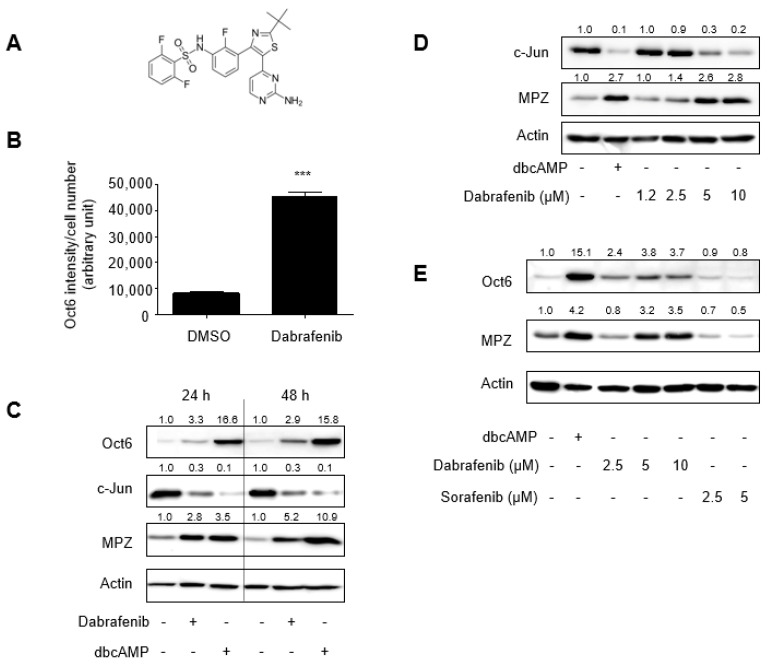
The identification of dabrafenib that promotes Schwann cell differentiation in vitro. (**A**) Chemical structure of dabrafenib. (**B**) Primary rat Schwann cells were treated with either DMSO (0.1%) or dabrafenib (10 µM) for 48 h and subjected to indirect immunofluorescence with an anti-Oct6 antibody. Wide-field fluorescence images from four fields in each well of a 384-well plate were acquired, and the fluorescence intensity of Oct6 expression divided by the number of cells is shown. Each column represents the mean ± SD, *n* = 3. *** *p* < 0.001, DMSO versus dabrafenib. (**C**) Primary rat Schwann cells were treated with either DMSO (0.1%), dabrafenib (10 µM), or dibutyryl cAMP (500 µM) for 24 or 48 h, and the total lysates were subject to western blot analyses. Band intensities were quantitated using ImageJ and normalized to actin intensities. Fold changes are indicated at the top of each lane. (**D**) The primary Schwann cells were treated with either DMSO (0.1%), dibutyryl cAMP (500 µM), or increasing concentrations of dabrafenib for 48 h, and the total lysates were subject to western blot analyses. (**E**) Primary rat Schwann cells were treated with either DMSO or the indicated chemicals for 48 h, and the total lysates were subject to Western blot analyses.

A library of commercially available synthetic small molecules was screened at a concentration of 10 µM for their ability to enhance Oct6 expression in primary Schwann cells, and we identified, among others, dabrafenib as a promising candidate (Figure 1A,B). Western blot analysis of total lysates derived from Schwann cells treated with either DMSO or dabrafenib confirmed that the expression of Oct6 and myelin protein zero (MPZ) was enhanced, whereas the expression of c-Jun was decreased in dabrafenib-treated cells compared with that in DMSO-treated cells (Figure 1C,D and Supplementary Figure S1). Dibutyryl cyclic AMP (dbcAMP) was used as a reference compound. The altered protein expression of differentiation biomarkers was further validated by comparing gene expression levels measured via microarray analysis. As shown in Table 1, several of the previously reported genes associated with Schwann cell differentiation, except for Sox10, Zeb2, and PAX3, were upregulated by dabrafenib treatment. These results, together with the phenotypic observation of Schwann cell using IncuCyte^®^ live-cell imaging (Supplementary Materials, avi files), demonstrated the ability of dabrafenib to induce Schwann cell differentiation in vitro.

Dabrafenib was developed as a Raf inhibitor exhibiting an IC_50_ below 10 nM in cell-free assays [11]. However, its effective concentration in Schwann cell differentiation assays was above 5 µM (Figure 1D). Therefore, our results raised the question as to whether dabrafenib-induced Schwann cell differentiation is mediated through Raf inhibition. To address this, we employed sorafenib, an inhibitor of Raf-1 and B-Raf with IC_50_ values of 6 nM and 22 nM in cell-free assays, respectively [12], and tested if sorafenib can also induce the expression of Oct6 and MPZ in rat primary Schwann cells. Western blot analysis revealed that sorafenib at concentrations up to 5 µM had no detectable effects on the expression of Oct6 or MPZ (Figure 1E). This suggests that Raf inhibition by dabrafenib is insufficient for driving Schwann cell differentiation or that the effects of dabrafenib on Schwann cell differentiation are mediated independently of Raf inhibition.

### 2.2. Mechanistic Insights into Dabrafenib-Induced Schwann Cell Differentiation

**Figure 2:**
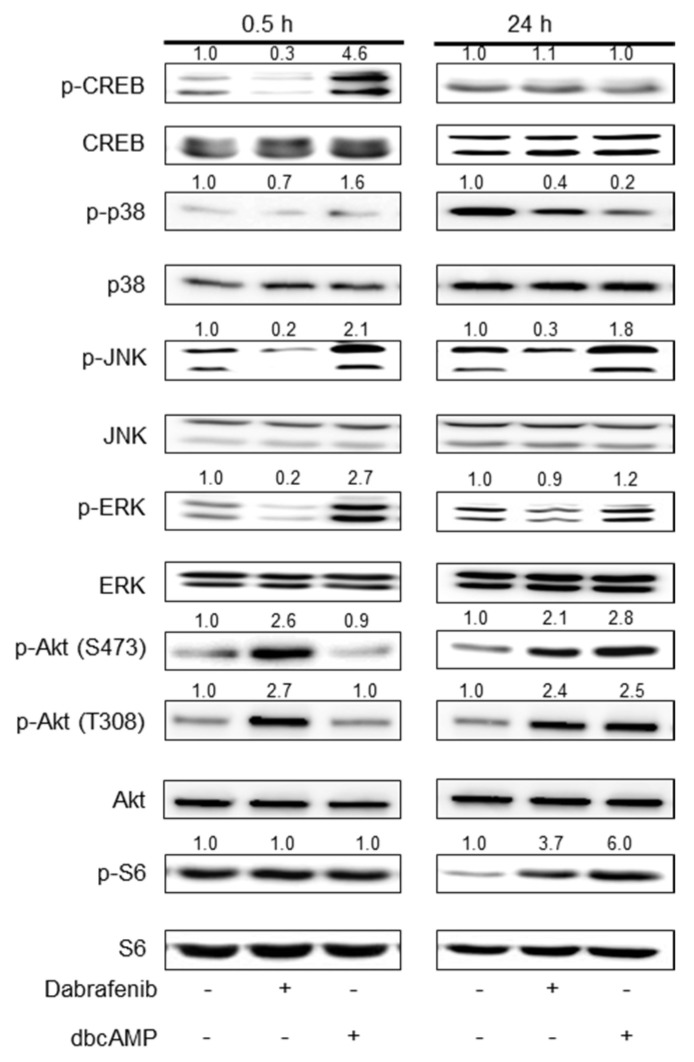
Analyses of signaling molecules in Schwann cells treated with dabrafenib. Primary rat Schwann cells were treated with either DMSO (0.1%), dabrafenib (10 µM), or dibutyryl cAMP (500 µM) for 0.5 or 24 h, and the total lysates were subject to Western blot analyses. Band intensities for phosphorylated proteins were quantified using ImageJ and normalized to total protein. Fold changes are indicated at the top of each lane.

To gain insight into how dabrafenib induces Schwann cell differentiation in vitro, we measured the phosphorylation levels of key signaling molecules implicated in Schwann cell differentiation. Primary rat Schwann cells were treated with dabrafenib (10 µM) for either 0.5 or 24 h, and the total cell lysates were subject to Western blot analysis. DMSO and dbcAMP were used as a negative control and reference compound, respectively. DbcAMP is a non-degradable analog of cAMP that stimulates cAMP-dependent protein kinases. It induces the phosphorylation of substrates, including cAMP response element binding protein (CREB), and reportedly induces Schwann cell differentiation in vitro [13]. In our study, CREB phosphorylation was dramatically increased by dbcAMP at 0.5 h; however, dabrafenib treatment resulted in a decrease in CREB phosphorylation (Figure 2). These results suggest that the effects of dabrafenib on Schwann cell differentiation are mediated independently of the cAMP signaling pathway.

We analyzed the phosphorylation of three mitogen-activated protein kinases and found that, in contrast to the dbcAMP-enhanced phosphorylation of JNK and ERK, dabrafenib inhibited the basal levels of JNK and ERK phosphorylation at 0.5 h (Figure 2 and Supplementary Figure S2). These observations also suggested that the activation of JNK and ERK, in addition to cAMP signaling, is not necessary for dabrafenib-induced Schwann cell differentiation.

The activation of the phosphatidylinositol-3-kinase (PI3K)-Akt signaling pathway is a key event downstream of the Nrg1 (type III)/ErbB2 interaction. Unexpectedly, we observed that Akt phosphorylation at Ser473 and Thr308 was dramatically enhanced by dabrafenib treatment at 0.5 h and persisted during in vitro differentiation (Figure 2 and Supplementary Figure S2). Although the Akt phosphorylation events at Ser473 and Thr308 were evident by treatment with dbcAMP at 24 h, dbcAMP had no detectable effect on Akt phosphorylation at 0.5 h.

### 2.3. Inhibition of JNK Activity Exhibits No Effect on Schwann Cell Differentiation

The observation that dabrafenib inhibited JNK phosphorylation in Schwann cells prompted us to test whether the inhibition of JNK activity contributed to dabrafenib-induced Schwann cell differentiation. The incubation of Schwann cells with a JNK inhibitor resulted in the inhibition of substrate phosphorylation by JNK (c-Jun at Ser63) at 30 min (Figure S3A) and the suppression of c-Jun expression after 48 h (Figure S3B). However, although dabrafenib inhibited JNK phosphorylation, dabrafenib had no effect on c-Jun phosphorylation at Ser63. In addition, a 48-h treatment of Schwann cells with the JNK inhibitor resulted in no effect on MPZ expression compared with its expression in DMSO-treated cells (Figure S3B). This indicates that the inhibition of JNK activity was not sufficient to induce myelin gene expression or played a minor, if any, role in Schwann cell differentiation in vitro.

### 2.4. PI3K Activity Is Required for Dabrafenib-Induced Schwann Cell Differentiation

**Figure 3:**
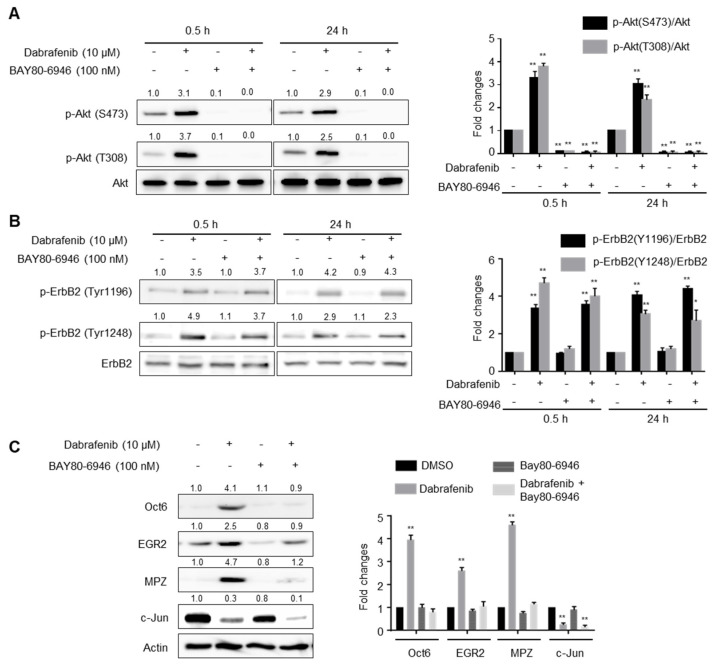
The effects of a PI3K inhibitor on dabrafenib-induced Schwann cell differentiation in vitro. (**A**) Primary rat Schwann cells were treated with the indicated compounds for 0.5 or 24 h, and the total lysates were subject to Western blot analyses. One representative Western blot is shown (left). Data from three independent replicates (mean ± S.D.) are shown as bar graphs (right). ** *p* < 0.01 versus control (DMSO). (**B**) Dabrafenib enhances ErbB2 autophosphorylation in primary Schwann cells. Primary rat Schwann cells were treated with the indicated compounds for 0.5 or 24 h, and the total lysates were subject to Western blot analyses. One representative Western blot is shown (left). Data from three independent replicates (mean ± S.D.) are shown as bar graphs (right). * *p* < 0.05 versus control (DMSO). ** *p* < 0.01 versus control (DMSO). (**C**) PI3K activity contributes to the dabrafenib-induced Schwann cell differentiation. Primary rat Schwann cells were treated with the indicated compounds for 48 h, and the total lysates were subject to Western blot analyses. One representative Western blot is shown (left). Data from three independent replicates (mean ± S.D.) are shown as bar graphs (right). ** *p* < 0.01 versus control (DMSO).

Because Akt phosphorylation is regulated by PI3K activity, we determined whether PI3K activity was required for dabrafenib-induced Akt phosphorylation. To test this, primary rat Schwann cells were treated for 0.5 or 24 h with dabrafenib in the absence or presence of a PI3K inhibitor, BAY80-6946. As shown in Figure 3A, Akt phosphorylation by dabrafenib was almost completely inhibited by BAY80-6946, indicating that PI3K activity is indeed required for Akt phosphorylation by dabrafenib.

Activation of PI3K-Akt pathway is preceded by ErbB2 autophosphorylation at Tyr1196 and Tyr1248 [14]. Therefore, we examined whether dabrafenib can induce autophosphorylation of ErbB2 in primary rat Schwann cells. The cells were untreated or treated with dabrafenib for 0.5 or 24 h, and the total cell lysates were subject to Western blot analysis. The results showed that autophosphorylation at Tyr1196 and Tyr1248 was significantly enhanced at 0.5 h by dabrafenib treatment and persisted during differentiation in vitro (Figure 3B). In contrast, dabrafenib-induced ErbB2 autophosphorylation was not affected by the PI3K inhibitor. These results suggest that dabrafenib-induced PI3K-Akt activation is a consequence of ErbB2 autophosphorylation.

We then determined whether PI3K activity contributed to the dabrafenib-induced expression of differentiation biomarkers. Primary rat Schwann cells were treated for 48 h with dabrafenib in the absence or presence of a PI3K inhibitor, BAY80-6946 [15], and the expression of each biomarker was analyzed by Western blot analysis. The results showed that dabrafenib treatment resulted in induced expression of Oct6, EGR2, and MPZ. Conversely, the expression was reduced by exposure to the PI3K inhibitor to levels observed in the DMSO control (Figure 3C). By contrast, the down-regulation of c-Jun expression by dabrafenib was not affected by the inhibition of PI3K (Figure 3C), indicating that pathways other than PI3K-Akt signaling are involved in dabrafenib-induced downregulation of c-Jun expression.

### 2.5. Dabrafenib Induces Autophosphorylation of ErbB2 by Inhibiting the MEK-ERK Pathway

**Figure 4:**
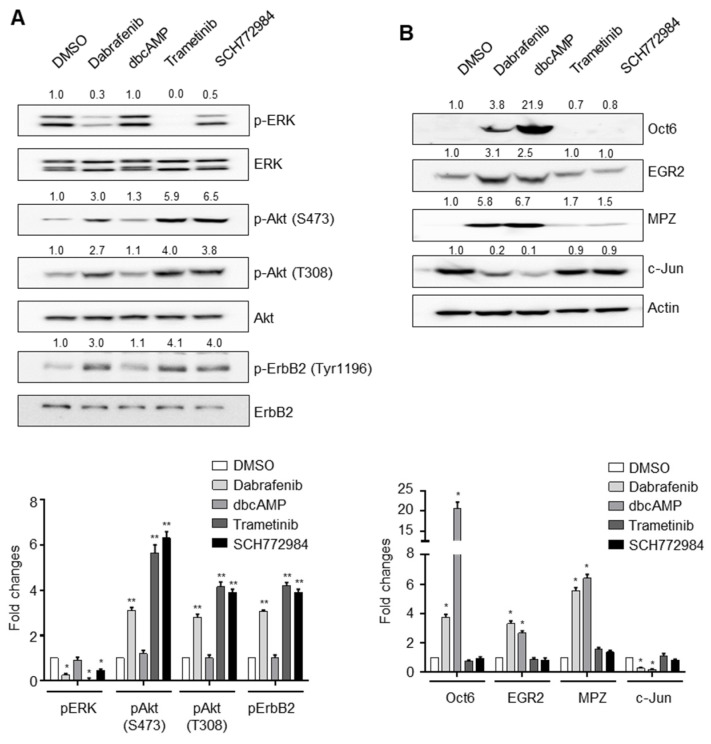
The effects of MEK/ERK inhibitors on ErbB2 signaling. (**A**) Inhibition of the MEK-ERK signaling pathway results in the activation of ErbB2-PI3K-Akt axis. Primary rat Schwann cells were treated with the indicated compounds for 0.5 h, and then total lysates were subject to Western blot analyses. One representative Western blot is shown (top). Data from three independent replicates (mean ± S.D.) are shown as bar graphs (bottom). ** *p* < 0.001 versus control (DMSO) * *p* < 0.01 versus control (DMSO). (**B**) Inhibition of MEK-ERK pathway is not sufficient to induce Schwann cell differentiation in vitro. Primary rat Schwann cells were treated with the indicated compounds for 48 h, and the total lysates were subject to Western blot analyses. One representative Western blot is shown (top). Data from three independent replicates (mean ± S.D.) are shown as bar graphs (bottom). * *p* < 0.01 versus control (DMSO).

We next evaluated the mechanism by which dabrafenib induces ErbB2 autophosphorylation at Tyr1196 and Tyr1248. Based on reports showing that ERK-mediated threonine phosphorylation of ErbB2 plays a role in the inhibition of ErbB2 [16] and our data demonstrating the ability of dabrafenib to inhibit ERK phosphorylation (Figure 2), we hypothesized that dabrafenib induces ErbB2 autophosphorylation by inhibiting the MEK-ERK signaling pathway. To assess this hypothesis, primary rat Schwann cells were treated with the small-molecule inhibitors of MEK and ERK, trametinib (0.03 µM) and SCH772984 (0.5 µM), respectively. The results indicated that, when compared with DMSO-incubated cells, MEK and ERK inhibitors enhanced the levels of ErbB2 autophosphorylation and Akt phosphorylation (Figure 4A) but had little effect, if any, on the expression of c-Jun, Oct6, EGR2, or MPZ after incubation for 48 h (Figure 4B). This suggests that the activation of the PI3K-Akt signaling pathway alone is not sufficient to induce Schwann cell differentiation in vitro.

Finally, we tested if dual inhibition of JNK and ERK activity could emulate the effects of dabrafenib. However, simultaneous treatment of Schwann cells with a JNK inhibitor (JNK inhibitor VIII) and an ERK inhibitor (trametinib) had little effect on the expression of Oct6, EGR2, or MPZ (data not shown).

## 3. Discussion

We identified dabrafenib as a pharmacological tool that promotes Schwann cell differentiation in vitro. Dabrafenib was developed as a small molecule inhibitor of Raf, which is a serine/threonine-specific protein kinase that lies upstream of the MEK-ERK signaling pathway. Raf inhibition by dabrafenib may contribute to the differentiation of Schwann cells in vitro because the inhibition of MEK and ERK activity led to an enhancement of ErbB2 autophosphorylation and Akt phosphorylation. However, the inhibition of the MEK-ERK axis was not sufficient to drive Schwann cell differentiation. Therefore, additional signaling pathways are likely responsible and should be identified as potential targets of dabrafenib in Schwann cells. In fact, dabrafenib at concentrations above 5 µM is expected to bind multiple kinases. (http://lincs.hms.harvard.edu/db/sm/10284-101).

The dual phosphorylation of threonine and tyrosine residues in the kinase subdomain VIII of JNK is correlated with its kinase activity [11,17]. However, the inhibition of JNK phosphorylation did not result in the inhibition of its target, c-Jun phosphorylation (Ser-63), in dabrafenib-treated Schwann cells. These results suggest that c-Jun phosphorylation at Ser-63 is not mediated by JNK (p46 or p54 kDa isoforms). Although it is beyond the scope of the present investigation, determining whether the p46 and p54 kDa isoforms exhibit different preferences toward phosphorylation sites would be important. Nonetheless, c-Jun expression was reduced by both dabrafenib and JNK inhibitor treatments in Schwann cells, and we speculate that the ability of dabrafenib to inhibit JNK phosphorylation may also contribute to driving Schwann cell differentiation.

The function and role of ERK in Schwann cell proliferation, differentiation, and myelination remains controversial. However, it appears that the outcomes following ERK activation or inhibition are different depending on the context in which ERK is engaged (i.e., in vitro, in vivo, or early versus later cellular developmental stage [18,19,20]). In cultured primary Schwann cells, a selective activation of the ERK pathway suppressed Schwann cell differentiation [21]. Syed et al. [22] demonstrated that treatment with a MEK inhibitor blocks the inhibitory function of ectopic, soluble Nrg1 and restores myelination. However, the mechanism through which MEK/ERK activation inhibits Schwann cell differentiation remains unclear. In our study, the inhibition of MEK or ERK by the small molecule resulted in the enhancement of ErbB2 autophosphorylation and Akt phosphorylation. These results suggest that the effects of dabrafenib on the phosphorylation of ErbB2 and Akt were phenocopied by pharmacological inhibition of the MEK-ERK pathway, indicating a negative control of ErbB2 by MEK-ERK.

Nrg1 reportedly exert diverse functions that differ depending on the developmental stage of Schwann cells [6]. Nrg1 is essential for the proliferation of Schwann cell precursors and also provides signals for myelination. In the study, we cultured primary Schwann cells for proliferation in the presence of soluble Nrg1 type III (30 ng/mL) in vitro. We observed that in the absence of Nrg1, dabrafenib-induced differentiation of Schwann cells requires ErbB2 (an Nrg1 receptor) and its downstream signaling activity. These observations raise the question as to how the activation of ErbB2 signaling mediates different outcomes (proliferation vs. differentiation) in Schwann cells. We propose that MEK-ERK signaling negatively regulates ErbB2 autophosphorylation, and our data provide insight into crosstalk between the MEK-ERK signaling pathway and the PI3K-Akt axis in Schwann cell biology.

In conclusion, by exploiting dabrafenib as a small-molecule tool to understand the molecular mechanisms regulating Schwann cell differentiation in vitro, we found that (1) proliferative progenitor-like Schwann cells can differentiate into cells expressing myelin genes in a manner independent of cAMP signaling, which was previously considered as a key signaling event in Schwann cell differentiation; (2) the PI3K-Akt signaling pathway is required, but not sufficient, to induce Schwann cell differentiation; (3) the expression of Oct6, MPZ, and EGR2 are regulated by mechanisms that differ from that of c-Jun as evidenced by the observation that the PI3K inhibitor abolished the up-regulation of Oct6, MPZ, and EGR2 by dabrafenib, but not the down-regulation of c-Jun by dabrafenib; and (4) MEK-ERK signaling negatively regulates ErbB2 autophosphorylation. Although additional molecules targeted by dabrafenib remain to be identified, our data provide insights into the crosstalk that exists between the MEK-ERK signaling pathway and the PI3K-Akt axis in Schwann cell differentiation.

## 4. Materials and Methods

### 4.1. Chemicals

Dabrafenib (purity > 99% by HPLC), sorafenib (purity > 99% by HPLC), JNK inhibitor VIII (purity > 99% by HPLC), trametinib (purity > 99% by HPLC), BAY80-6946 (purity > 99% by HPLC), and SCH772984 (purity > 99% by HPLC) were purchased from Selleckchem (Houston, TX, USA). N^6^,2′-O-Dibutyryl adenosine 3’,5′-cyclic monophosphate (purity > 96% by HPLC) and forskolin (purity > 98% by HPLC) were purchased from Merck (Kenilworth, NJ, USA).

### 4.2. Antibodies

The antibody against Oct6 was purchased from Santa Cruz (Dallas, TX, USA). Antibodies against myelin protein zero (MPZ), EGR2, and phospho-ErbB2 (Tyr1196) were purchased from Abcam (Cambridge, MA, USA). All other antibodies, including c-Jun, CREB, phospho-CREB (Ser133), JNK, phospho-JNK (Thr183/Tyr185), ERK1/2, phospho-ERK1/2 (Thr202/Tyr204), S6, phospho-S6 (Ser240/244), p38, phospho-p38 (Thr180/Tyr182), Akt, phospho-Akt (Ser473), phospho-Akt (Thr308), ErbB2, and phospho-ErbB2 (Tyr1248), were purchased from Cell Signaling Technology (Danvers, MA, USA).

### 4.3. Primary Rat Schwann Cell Culture and Chemical Treatment

Primary Schwann cells were prepared from the sciatic nerves of 4-day-old Sprague-Dawley rats as previously described [23]. The cells were cultured in DMEM containing 1% FBS, 30 ng/mL of Nrg1 (human NRG1-β1 extracellular domain, R&D Systems), N-2 supplement (Gibco, Waltham, MA, USA)) and 5 µM forskolin for two generations. Then, the cells were seeded onto either 384-well plates at a density of 7 × 10^3^ cells/well or 10-cm dishes at a density of 2 × 10^5^ cells/dish in DMEM containing 1% FBS, 30 ng/mL Nrg1, N-2 supplement, and 5 µM forskolin. After a 24-h incubation, the culture medium was changed to DMEM containing 1% FBS, and then either DMSO (0.1%) or compound was added. The cells were incubated for 0.5–48 h and subjected to either indirect immunofluorescence, Western blot analysis, or total RNA isolation.

### 4.4. Indirect Immunofluorescence and Oct6 Quantitation

The fixed cells were washed with PBS and permeabilized with 0.25% Triton X-100 in PBS for 8 min. The primary and secondary antibodies were diluted in PBS containing 5% bovine serum albumin. The antibodies were as follows: 0.2 µg/mL of goat anti-Oct6 and 2 µg/mL of Alexa Fluor 594 rabbit anti-goat IgG. To measure the intensity of Oct6 expression, wide-field fluorescence images from four fields in each well of the 384-well plate were acquired with the ArrayScan VTI HCS Reader (Thermo Fisher Scientific, Waltham, MA, USA) using a 10× objective lens. The images were analyzed with the target activation V3 BioApplication (Thermo Fisher Scientific). 4′,6′-Diamidino-2-phenylindole was used to stain the nucleus.

### 4.5. Western Blot Analysis

The cells were washed with PBS, collected in a RIPA buffer, incubated at 4 °C for 20 min, and then centrifuged at 13,000 rpm for 10 min. An equal amount of each sample was resolved on an SDS-polyacrylamide gel and electroblotted onto a PVDF membrane. The blots were blocked with 5% skim milk in PBS containing 0.05% Tween 20 and incubated with an appropriate antibody (diluted 1:1000 in blocking solution). Immobilon Western HRP substrate (Merck, Kenilworth, NJ, USA) and ImageQuant LAS 4000 (GE Healthcare) were used to detect chemiluminescence signals. Band intensities were quantified using ImageJ software and normalized to actin intensities. One representative Western blot for each experiment is shown in the figures, and fold changes in band intensity were calculated against a control and indicated at the top of each lane. Data from two or three independent experiments were averaged for each condition and presented in the form of bar graphs. Data are expressed in fold changes compared with control (mean ± S.D.).

### 4.6. Statistical Analysis

Statistical analysis was performed using GraphPad Prism, version 6.07, and *p* values were calculated by paired t test. *p*-values of <0.05 were considered statistically significant.

### 4.7. Microarray Analysis

Total RNA was isolated, amplified, and converted to antisense RNAs for analysis. Microarray experiments were performed using the GeneChip Rat Gene 2.0 ST Array (Affymetrix), and data were acquired using the GeneChip Scanner 3000DX. Gene-level analyses were conducted using the Affymetrix Transcriptome Analysis Console v4.0 software. The resulting CEL files were processed using RMA normalization. Differential expression between samples was predicted at the gene level. An unpaired t-test was used to compare individual gene expression data. Differentially expressed genes were defined based on an absolute fold change equal to or greater than 2.0 and a *p*-value less than or equal to 0.05. The absolute fold change (Dabrafenib versus DMSO) values for each gene were plotted onto X (1st experiment) and Y (2nd experiment) axis on a graph (Supplementary Figure S4).

## Figures and Tables

**Table 1:** Genes associated with Schwann cell differentiation.

Gene	Fold Changes (Mean ± SD, *n* = 2)
Myelin Basic Protein	5.48 ± 0.72
Myelin Protein Zero	4.30 ± 0.14
Early Growth Response 2	5.13 ± 0.58
Myelin Associated Glycoprotein	3.47 ± 0.24
Oct6 (Pou3f1)	3.08 ± 0.84
Brn2 (Pou3f2)	2.01 ± 0.01
Sox10	1.01 ± 0.01
Zeb2	1.02 ± 0.03
PAX3	1.01 ± 0.01
c-Jun	−4.85 ± 0.63

## Data Availability

Data are contained within the article and Supplementary Materials.

## Supplementary Materials

The following are available online, Figures S1–S4. Video S1: DMSO, Video S2: Dabrafenib, Video S3: dbcAMP.

## Sample Availability

Samples are not available from the authors.
