# Supplementary material for: Dabrafenib Promotes Schwann Cell Differentiation by Inhibition of the MEK-ERK Pathway
Source: Molecules. 2021 Apr 8;26(8):2141. doi: 10.3390/molecules26082141 (PMC8068149; doi:10.3390/molecules26082141)
Supplement: Supplementary file 1 [file molecules-26-02141-s001.pdf]

Supplementary Information

Dabrafenib promotes Schwann cell differentiation by inhibition of the MEK-ERK pathway

Kyuhee Park[1], Yoon Kyoung Shin[2], Gyeongbeen Lee[1], Hwan Tae Park[2], and Yongmun Choi[1*]

[1]Biocenter, Gyeonggido Business and Science Accelerator, Suwon 16229, Korea

[2]Department of Physiology, Peripheral Neuropathy Research Center, Donga University Medical School, Busan 49201, Korea

* Corresponding author: Yongmun Choi

E-mail, ychoi@gbsa.or.kr.

Materials and Methods

Primary rat Schwann cell culture and chemical treatment

Primary Schwann cells were prepared from the sciatic nerves of 4-day-old Sprague-Dawley rats. The cells were cultured in DMEM containing 1% FBS, 30 ng/ml of Nrg1 (human NRG1-β1 extracellular domain, R&D Systems), N-2 supplement (Gibco), and 5 μM forskolin for two generations. Then, the cells were seeded into 10-cm dishes at a density of 2 x 10^5 cells/dish in DMEM containing 1% FBS, 30 ng/ml of Nrg1, N-2 supplement, and 5 μM forskolin. After a 24-h incubation, the culture medium was changed to DMEM containing 1% FBS, and then either DMSO (0.1%) or dabrafenib (10 μM) was added. The cells were incubated for 48 h, and live cell images were acquired using IncuCyte Live-Cell Analysis System (Essen BioScience).

Western blot analysis

The cells were washed with PBS, collected in RIPA buffer, incubated at 4 °C for 20 min, and then centrifuged at 13,000 rpm for 10 min. Equal amount of the samples were resolved on a SDS-polyacrylamide gel and electroblotted onto a PVDF membrane. The blots were blocked with 5% skim milk in PBS containing 0.05% Tween 20 and incubated with an appropriate antibody (diluted 1:1000 in blocking solution). Immobilon Western HRP substrate (Merck) and ImageQuant LAS 4000 (GE Healthcare) were used to detect chemiluminescence signals. Band intensities were quantitated using Image J software and normalized to actin intensities. The data from two independent experiments were averaged for each condition and presented in the form of bar graphs. Data are expressed as a fold change compared with control (mean ± S.D.).

Supplementary Figures and Legends

Figure S1.

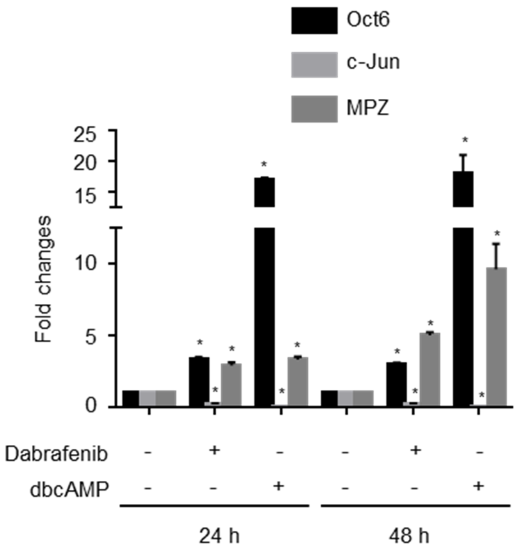

Figure S1. The effects of dabrafenib on the expression of Oct6, c-Jun, and MPZ. Primary rat Schwann cells were treated with either DMSO (0.1%), dabrafenib (10 μM), or dibutyryl cAMP (500 μM) for 24 or 48 h, and the total lysates were subject to western blot analyses. The data from two independent experiments were averaged for each condition and presented in the form of bar graphs. *$p < 0.05$ versus non-treated control (DMSO).

Figure S2

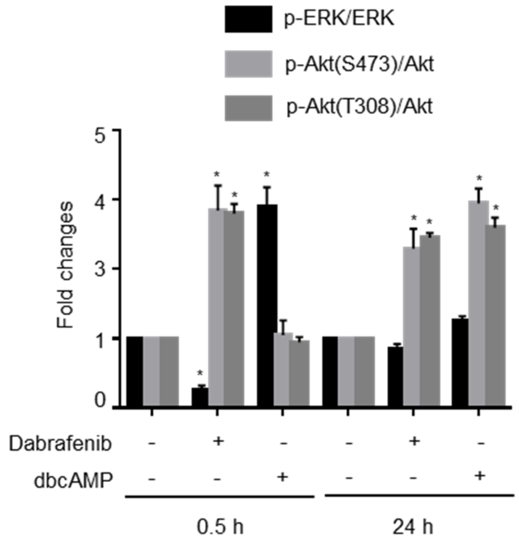

Figure S2. Analyses of signaling molecules in Schwann cells treated with dabrafenib. Primary rat Schwann cells were treated with either DMSO (0.1%), dabrafenib (10 μM), or dibutyryl cAMP (500 μM) for 0.5 or 24 h, and the total lysates were subject to western blot analyses. The data from two independent experiments were averaged for each condition and presented in the form of bar graphs. *p < 0.05 versus non-treated control (DMSO).

Figure S3

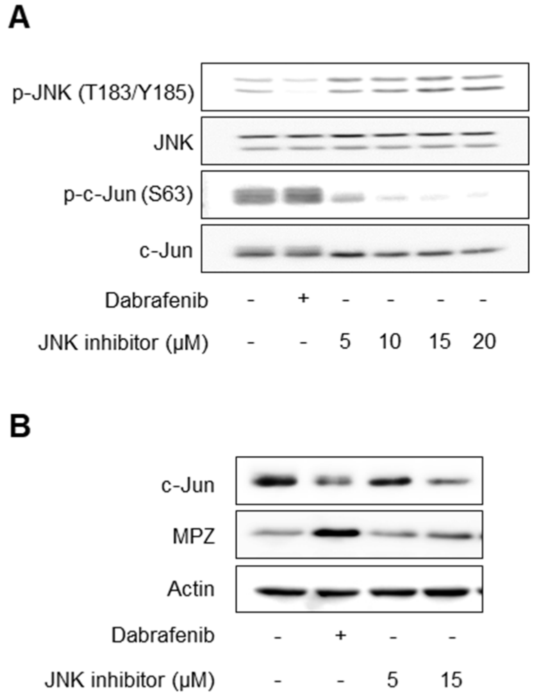

Figure S3. The effects of JNK inhibitor VIII on Schwann cell differentiation. Primary rat Schwann cells were treated with either DMSO (0.1%), dabrafenib (10 µM), or JNK inhibitor VIII for 0.5 (A) or 48 h (B), and the total lysates were subject to western blot analyses.

Figure S4

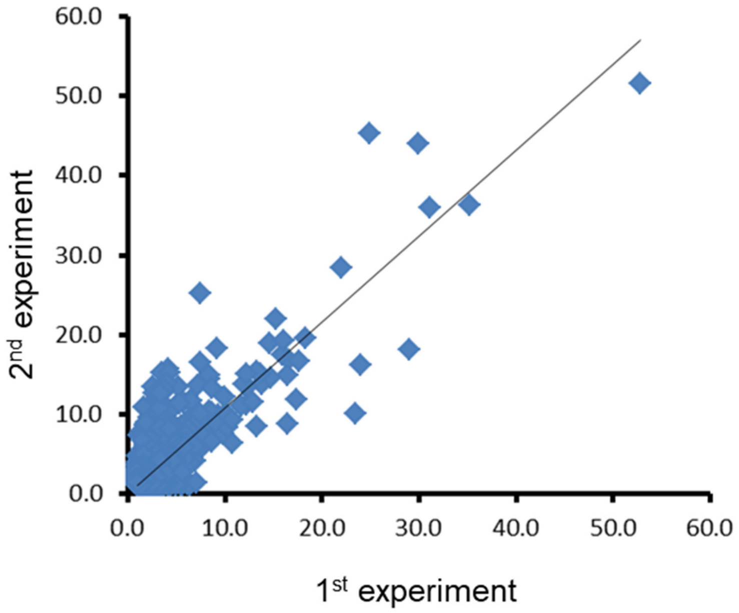

Figure S4. Scatter plot of absolute fold changes (FCAbsolute, Dabrafenib versus DMSO) derived from two independent experiments.
